# Diet-Induced Metabolic Dysfunction of Hypothalamic Nutrient Sensing in Rodents

**DOI:** 10.3390/ijms23073958

**Published:** 2022-04-02

**Authors:** Isabel Arrieta-Cruz, Blanca Samara Torres-Ávila, Hilda Martínez-Coria, Héctor Eduardo López-Valdés, Roger Gutiérrez-Juárez

**Affiliations:** 1 Department of Basic Research, National Institute of Geriatrics, Ministry of Health, Mexico City 10200, Mexico; iarrieta@inger.gob.mx; 2 Social Service Program, School of Medicine, Faculty of Higher Studies Zaragoza, National Autonomous University of Mexico, Mexico City 09230, Mexico; 15mc01.samaratorres@gmail.com; 3 Department of Physiology, Faculty of Medicine, National Autonomous University of Mexico, Mexico City 04510, Mexico; hildamcoria@gmail.com (H.M.-C.); helopezv@gmail.com (H.E.L.-V.); 4 Department of Biomedical Sciences, School of Medicine, Faculty of Higher Studies Zaragoza, National Autonomous University of Mexico, Mexico City 09230, Mexico

**Keywords:** nutrients, amino acids, mediobasal hypothalamus, glycemia, liver, high-fat diet, diabetes

## Abstract

A sedentary lifestyle and excessive nutrient intake resulting from the consumption of high-fat and calorie-rich diets are environmental factors contributing to the rapid growth of the current pandemic of type 2 diabetes mellitus (DM2). Fasting hyperglycemia, an established hallmark of DM2, is caused by excessive production of glucose by the liver, resulting in the inability of insulin to suppress endogenous glucose production. To prevent inappropriate elevations of circulating glucose resulting from changes in nutrient availability, mammals rely on complex mechanisms for continuously detecting these changes and to respond to them with metabolic adaptations designed to modulate glucose output. The mediobasal hypothalamus (MBH) is the key center where nutritional cues are detected and appropriate modulatory responses are integrated. However, certain environmental factors may have a negative impact on these adaptive responses. For example, consumption of a diet enriched in saturated fat in rodents resulted in the development of a metabolic defect that attenuated these nutrient sensing mechanisms, rendering the animals prone to developing hyperglycemia. Thus, high-fat feeding leads to a state of “metabolic disability” in which animals’ glucoregulatory responses fail. We postulate that the chronic faltering of the hypothalamic glucoregulatory mechanisms contributes to the development of metabolic disease.

## 1. Introduction

The increasing prevalence of type 2 diabetes mellitus (DM2) and obesity in recent years is strongly linked to a lifestyle characterized by excessive intake of nutrients and low physical activity [1,2]. Diabetes mellitus is defined as “a group of metabolic diseases characterized by hyperglycemia resulting from defects in insulin secretion, insulin action, or both” [3]. Hyperglycemia, a landmark of DM2, is caused by a faltering of the regulatory circuits that maintain circulating glucose within the normal range. In mammalian organisms, maintaining a narrow and steady level of blood glucose (euglycemia) is a basic requirement for the preservation of metabolic health between alternating periods of feeding and fasting, as well as when facing physical or psychological stress and other environmental challenges. There are two sources of glucose that can influence its circulating levels in physiological conditions: (i) food intake or (ii) hepatic glucose production; either glucose source can cause fluctuations in the level of plasma glucose and, consequently, set off the various mechanisms designed to maintain euglycemia. However, under the influence of certain environmental conditions, these glucoregulatory mechanisms may not work properly. For example, a crucial factor is the intake of calorie-rich and high-fat diets in Western societies, which can cause a marked increase in the influx of nutrients and prompt a series of adaptive responses, including changes in feeding behavior and nutrient metabolism aimed to prevent glycemic alterations beyond an individual’s set point [4,5,6].

The maintenance of glucose homeostasis relies on complex regulatory mechanisms that require the participation of several organs and tissues, including the pancreas, liver, skeletal muscle, intestine, adipose tissue, and the central nervous system (CNS) [5,7]. In this work, we focus on the role of the CNS with an emphasis on hypothalamic nutrient sensing and we summarize how overnutrition may lead to its dysfunction. In turn, this disturbance may set the stage for the development of metabolic dysfunction leading to blood glucose dysregulation which may eventually contribute to the onset of hyperglycemia, a crucial component of metabolic diseases such as DM2.

The literature cited in this work was selected after performing searches on PubMed from the National Library of Medicine of the National Institutes of Health (USA) Original articles, reviews, systematic reviews, and meta-analysis were included in the search using mainly the terms “high-fat diet”, “diet-induced insulin resistance”, and “hypothalamic nutrient sensing”. We focused on articles reporting studies that shed light on how HFD feeding affected metabolic nutrient sensing in animal models or human studies. Mechanistic studies on the topic were scarce, especially recent ones. Studies in humans that provided support for the concepts discussed were also included.

## 2. Role of the Hypothalamus in the Control of Glycemia

The mediobasal hypothalamus (MBH) of the brain gathers information about the body’s nutritional status to implement appropriate behavioral and metabolic responses to changes in nutrient availability [4,5,6,8]. This information is relayed to the MBH through at least two ways [9]: (1) changes in the levels of hormones (such as insulin or leptin) that are controlled by nutrient availability and (2) changes in circulating macronutrients or their metabolites. First, we focus on the latter mechanism, in which these macronutrients or some of their metabolites need to reach the MBH, where they are metabolically converted to certain key metabolites (described in detail below) to activate the glucoregulatory brain-liver circuit. This mechanism is called metabolic nutrient sensing. Numerous studies have established that the MBH is the brain area where metabolic nutrient sensing regulates systemic glucose or lipid metabolism [8,9,10]. It is widely accepted that blood metabolites can cross the blood brain barrier (BBB) by taking advantage of the distinctive microvasculature around the MBH [11], specifically, the arcuate nucleus (ARC) where neuronal populations are located which play important roles in the control of energy and glucose metabolism. The subependymal plexus, irrigating exclusively the ventromedial ARC from the subadjacent neuroendocrine median eminence, is equipped with fenestrated endothelial cells that make it highly permeable to many blood components, unlike many other parts of the BBB [11]. Thanks to this anatomical feature, macronutrients, as well as other circulating compounds, may easily gain access to the MBH.

In the MBH, macronutrients are metabolically converted to acetyl-CoA and malonyl-CoA [9,12]. Studies in the skeletal muscle of rodents have linked malonyl-CoA to the switching of fuel utilization from fatty acids oxidation to glucose oxidation. Furthermore, malonyl-CoA has been recognized to be a potent allosteric inhibitor of carnitine palmitoyltransferase 1 (CPT1), an enzyme that regulates the uptake of long-chain fatty acyl (LCFA)-CoA into the mitochondria [13]. Moreover, it was later recognized that malonyl-CoA acted as the fuel sensor responsible for the transition from fatty acids to glucose oxidation [14]. Indeed, in the presence of high levels of glucose and insulin, the buildup of malonyl-CoA inhibits CPT1, therefore, decreasing lipid oxidation and stimulating lipid storage into triacylglycerides (TAG). Interestingly, the intrahypothalamic administration of oleic acid has been demonstrated to inhibit food intake and glucose production in rodents [15]. This effect was not replicated by fatty acids of medium-chain length, which meant that oleic acid was acting as a distinct signal of nutrient abundance and that the actions of oleic acid were not just a consequence of its oxidation. In agreement with this concept, studies in rodents have shown that hypothalamic increases of endogenous LCFA-CoAs also signaled nutrient abundance [16]. Under physiological conditions, high levels of malonyl-CoA resulting from an elevated influx of nutrient-derived carbons may inhibit CPT1 activity. This high availability of LCFA-CoAs, then, triggers a central lipid sensing circuit that carries a neural signal to the liver, thus, reducing unnecessary endogenous nutrient output [9,16].

**Figure 1:**
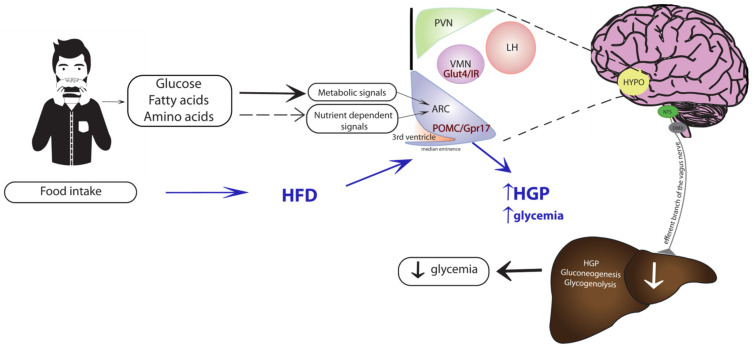
Brain-liver circuit for the control of glucose metabolism and food intake, and the effects of high-fat diet feeding. Macronutrients from diet act in the ARC neurons of the MBH through two main mechanisms: (1) direct nutrient metabolic signaling (black arrows) or (2) nutrient-dependent hormonal signaling (black dashed arrows). The mechanisms both lead to the generation of a neurogenic signal that is relayed to the liver via the efferent branch of the vagus nerve, eventually reducing glucose production (HGP) to maintain glucose homeostasis (black arrows). A potential role for Gpr17-expressing POMC neurons and neurons co-expressing IR and Glut4 (red) in the MBH after HFD feeding is depicted (blue arrows). ARC, arcuate nucleus; VMN, ventromedial nucleus; PVN, paraventricular nucleus; LH, lateral hypothalamus; HYPO, hypothalamus; NTS, nucleus tractus solitaries; DMX, dorsal motor nucleus of the vagus; POMC, pro-opiomelanocortin neurons; Gpr17, G protein-coupled receptor; Glut4, glucose transporter 4; IR, insulin receptor; HFD, high-fat diet.

Based on the abovementioned studies, it can be concluded that all major classes of macronutrients (fatty acids, glucose, and amino acids) can potentially act as metabolic nutrient signals in the MBH, since their metabolism leads to the production of acetyl- and/or malonyl-CoA (Figure 1) [12,14,17,18]. In fact, our groups and others have shown that members of all the major classes of macronutrients can engage the hypothalamic sensing mechanism to modulate the production of glucose by the liver [10,19,20,21,22]. In this work, we focus on the glucoregulatory action of amino acids (derived from dietary protein) and on how a diet enriched in saturated fat (the so-called Western diet) disrupts their glucoregulatory action(s). Lastly, we discuss the potential contribution of this acquired disruption of metabolic nutrient sensing to the development of hyperglycemia and metabolic disease.

## 3. Circulating Amino Acids in the Regulation of Glucose Production

As stated earlier, amino acids such as leucine and proline engage hypothalamic circuits of nutrient sensing to initiate a program of metabolic adjustments in the liver that modulate the rate of endogenous glucose production. We have previously shown that leucine is metabolized in the brain to acetyl-CoA and malony-CoA [22]; malony-CoA plays a decisive role in metabolic homeostasis and metabolic nutrient sensing [11,22]. Activation of hypothalamic “sensing” networks generates a response to acutely inhibit food intake, as well as glucose and lipid output by the liver. This response depends on the accumulation of malonyl-CoA in the MBH [22,23], which then leads to the production of oleyl-CoA. It has been proposed that oleyl-CoA activates the potassium sensitive ATP (K_ATP_) channels that, in turn, generate a neural signal, which is first relayed to the brainstem from where it travels to the liver via the vagus nerve [24]. Examples of this kind of regulatory mechanism include the modulation of hepatic glucose metabolism and triglyceride secretion by oleic acid, glucose, or lactate [10,15,19] and the amino acids: leucine, proline, isoleucine, and valine [22,25,26]. Interestingly, studies have shown that interventions that interrupted critical stages of the brain-liver circuit also disrupted the nutrient-driven hypothalamic regulation of liver glucose production [21]. Other studies have also shown regulation of hepatic glucose production by different amino acids such as histidine, but their postulated mechanisms responsible for their action were different from those discussed here [27]. Consequently, we have not discussed them further. Moreover, although in our studies with leucine we implicated acetyl-CoA, experiments with rapamycin have indicated that the central effects of leucine were mTOR independent. Interestingly, a recent report indicated that leucine could directly activate mTORC1 via its metabolite, acetyl-CoA [28]. Studies in rodents have shown that oral leucine supplementation improved glucose metabolism, further supporting a physiological role for leucine in the regulation of glycemia [29,30].

**Figure 2:**
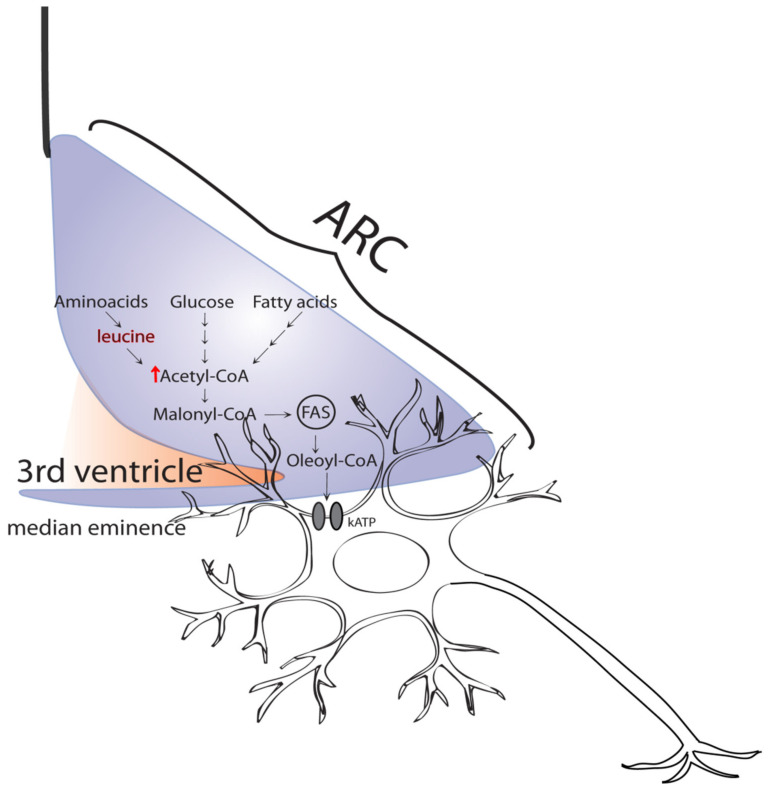
Postulated biochemical mechanisms of metabolic nutrient sensing in neurons of the arcuate nucleus (ARC) for the regulation of glycemia. Nutrients, such as leucine, are metabolized to acetyl-CoA and malonyl-CoA. Next, malonyl-CoA is utilized to produce oleoyl-CoA which, in turn, is proposed to activate hypothalamic K_ATP_ channels generating neurogenic signals that reach the liver via its vagal innervation. FAS, fatty acid synthase.

It is known that several amino acids can be metabolized to acetyl-CoA, the precursor of malonyl-CoA, consequently, several amino acids or their metabolites could be involved in the central regulation of glucose production (Figure 2). In fact, we have demonstrated that this was the case for leucine [22], proline [25], isoleucine, and valine [26]. As outlined above, these amino acids have free access to neurons in the MBH and can be taken up and metabolized by these cells [31]. It is well known that the various neuronal groups present in the ARC of the MBH are responsible for the regulation of glucose metabolism and energy balance [5,32]. In the ARC, there are several populations of neurons that possess metabolic regulatory roles, mainly the agouti-related peptide (AGRP) and proopiomelanocortin (POMC) neurons. These names are derived from the specific neuropeptides they produce that themselves participate in the regulatory processes that control energy balance and glucose metabolism. For example, recent studies have indicated that AGRP neurons were mainly involved in the control of glucose metabolism, while POMC neurons regulated energy balance [32]. Furthermore, other studies have indicated that the central actions of insulin on glucose metabolism were mediated by AGRP neurons [33]. Although these findings strongly suggest that metabolic nutrient sensing occurs in AGRP neurons, more direct evidence is required. Interestingly, recent data by our group suggested that, in some cases, other cell types such as astrocytes in combination with neurons may also be involved [25]. Despite these recent clues, limited information is currently available about the intricacies of the neuro-anatomical basis of metabolic nutrient sensing. Interestingly, the neuronal populations implicated in another nutrient sensing circuit, the gut-brain axis, have been recently characterized [34]. In this case, as predicted, distinct neuronal populations were required for regulation of glucose metabolism and food intake via nutrient sensing through the gut-brain axis. This does not mean that the same neuronal populations must be implicated in the brain-liver axis discussed in this work. Thus, unveiling the cellular basis of metabolic nutrient sensing in the hypothalamus will be the next major challenge of future studies.

## 4. Physiological Relevance of Metabolic Nutrient Sensing

Detailed studies on the characterization of the hypothalamic events underlying the ability of macronutrients to centrally regulate the metabolism of glucose in the liver have been carried out through the experimental modulation of the availability of these nutrients in the brain. In this regard, the most important question is if the glucoregulatory actions of macronutrients is physiologically relevant. To answer this question, we performed experiments to gain insight into the physiological relevance of metabolic nutrient sensing and its possible contribution to disease development in the liver of conscious animals. Thus, we performed systemic infusions of the amino acids leucine or proline to produce moderate elevations of the circulating levels of either one, while monitoring glucose metabolism during pancreatic clamp studies [22,25]. In these studies, acutely increasing the availability of either leucine or proline produced a marked decrease in circulating glucose, secondary to reduced HGP (Figure 1). Interestingly, inhibiting the metabolism of these amino acids in the hypothalamus with appropriate interventions markedly attenuated the glucoregulatory effect of increases in circulating proline or leucine. These results strongly suggest the physiological relevance of hypothalamic amino acid sensing in the control of glucose metabolism. In separate experiments in rats, we performed molecular interventions (such as overexpression of branched-chain ketoacid dehydrogenase kinase (BCKDK) in the MBH) designed to cripple leucine sensing, which made the animals susceptible to developing hyperglycemia when challenged with a protein-rich test meal [22]. This result indicated that disruption of central leucine sensing could be involved in the onset of metabolic disease.

According to the work summarized above, we developed the schematic shown in Figure 2, showing the metabolic pathways proposed to participate in the hypothalamic sensing of amino acids. Briefly, circulating leucine and proline are metabolized in the MBH to produce two common crucial metabolites: acetyl-CoA and malonyl-CoA. The latter (malonyl-CoA) is utilized to synthesize oleoyl-CoA, which is postulated to, then, activate hypothalamic K_ATP_ channels [35]. Activation of these channels is an early event in the generation of a neurogenic signal that reaches the liver via the hepatic branch of the vagus nerve. This vagal inflow changes glucose metabolic fluxes in the liver that, next, reduce glucose production, consequently, lowering circulating blood glucose. This flux redistribution includes a decrease in the rates of both gluconeogenesis and glycogenolysis, which together lead to a decrease in the total glucose output into the circulation, and therefore, lead to lower circulating glucose levels. This regulatory mechanism is very active postprandially, when the exogenous nutrient influx produces an increase in the circulating levels of macronutrients, including amino acids, thus, decreasing the demand for endogenous (hepatic) production of nutrients such as glucose. Of course, this liver-brain circuit is proposed to work in concert with other organ systems to prevent excessive postprandial increases in circulating glucose (Figure 1).

## 5. Disruption of Central Glucose Regulation by High-Fat Diets

**Figure 3:**
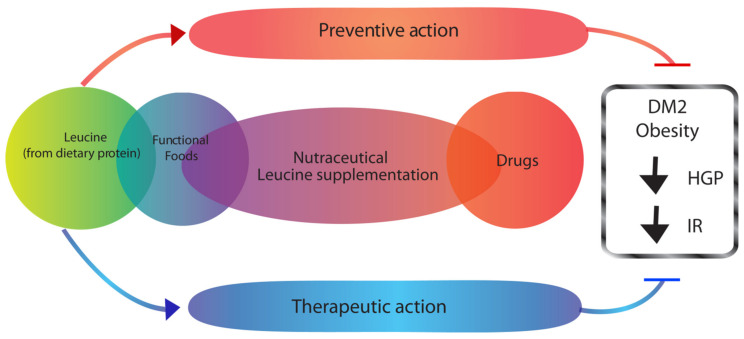
Potential nutraceutical use of nutrients, such as leucine derived from dietary protein or supplementation, for the prevention and/or treatment of diet-induced metabolic disability. HGP, hepatic glucose production; IR, insulin resistance; DM2, type 2 diabetes mellitus.

The consumption of calorie-rich diets that are high in saturated fat is a very important environmental factor contributing to the emergence and worsening of the current pandemic of diabetes and obesity [36,37]. Early studies have shown that rodents subjected to three days of feeding with a diet enriched in animal fat (high-fat diet) induced insulin resistance affecting mainly the liver. Interestingly, it was later shown that, under these conditions, the hypothalamic glucoregulatory response to fatty acids was markedly reduced [19,38,39]. These studies clearly indicated that an acquired defect that interfered with brain nutrient sensing was induced by the consumption of a diet enriched in saturated fat. Further studies have established that the hypothalamic sensing of glucose and lactate were also attenuated after high-fat diet (HFD) feeding [40]. Lastly, recent studies have shown that the central glucoregulatory action of leucine was markedly attenuated by high-fat diet-induced insulin resistance [26]. Thus, the central sensing of all three major classes of macronutrients (carbohydrates, lipids, and amino acids) is disrupted by short-term insulin resistance induced by high-fat feeding. Clearly, all the studies highlighted that consumption of a high-fat diet acutely produced a marked disruption in centrally mediated nutrient sensing. However, what is the mechanism(s) underlying this form of acquired “metabolic disability” that renders the organism unable to respond normally to nutrient cues? The answer to this question is largely unknown; however, studies in rats [39] have led to two relevant findings: (1) HFD feeding caused a marked decrease in the levels of hypothalamic long-chain fatty acyl CoAs (LCFA-CoA); and (2) restoration of the hypothalamic LCFA-CoA pool normalized nutrient-dependent glucose regulation. These metabolic deficits were in line with our previous studies that indicated the central metabolism of nutrients in the hypothalamus required an increase in the local levels of oleoyl-CoA [22]. In fact, early studies had already shown that oleoyl-CoA could activate K_ATP_ channels of the same type found in the brain [35]. Upon activation, hypothalamic K_ATP_ channels subsequently generated the neurogenic signal required to modulate liver glucose metabolic fluxes (Figure 2). Importantly, these observations further suggest that reducing the content of saturated fat in the diet would be helpful for preventing or delaying the development of defective nutrient sensing, thus, preserving normal postprandial glycemic control. Clearly, uncovering the mechanistic details of how high-fat feeding produces this putative “metabolic disability” will be necessary for designing creative interventions aimed at preventing or even reverting the metabolic unresponsiveness accompanying moderate insulin resistance. Diets that limit the consumption of saturated fats would be a key factor in preventing or even reverting, the disruption of nutrient-driven regulation of liver glucose production. This could be accompanied by enrichment of the diet with certain macronutrients, for example, fish and plant protein or even specific amino acids or sets of them (Figure 3). Accordingly, recently, controlled studies in humans, as well meta-analysis reviews of studies in humans, have shown several positive actions of high-protein diets in patients with diabetes, including improved glycemic control, improved insulin sensitivity, and reduced body adiposity [41,42,43]. Furthermore, we postulate that restoring the levels of key hypothalamic molecules implicated in the sensing mechanism(s) such as malonyl-CoA or oleyl-CoA would be an important avenue of research. In fact, studies in rodents have suggested that restoring the levels of long-chain fatty acyl-CoAs in the hypothalamus could be helpful to overcome the metabolic dysfunctions brought about by high-fat diets [39]. A nutraceutical candidate to achieve this restoration is leucine itself, since it is a ketogenic amino acid that improves insulin action [22]; we postulate that leucine could be a dietary supplement to overcome the damaging effects of chronic HFD feeding. In support of this hypothesis, a recent study in mice [44] showed that a simultaneous intervention of exercise and leucine supplementation increased systemic insulin sensitivity by reducing liver and adipose tissue inflammation through a decrease in NF-κB p65 phosphorylation and an increase in the expression of adiponectin in adipose tissue. Thus, the development of pharmacological molecules designed to potentiate central nutrient sensing could be a promising treatment alternative, in addition to high protein dietary approaches.

In addition to the concepts discussed above, another recent development is that studies in rodents have shown that the chronic consumption of diets high in saturated fats induced inflammation oxidative stress in the hypothalamus [45,46,47]. This inflammatory response was characterized by hypothalamic increases in oxidative markers including reactive oxygen species (ROS), malondialdehyde (MDA), superoxide dismutase (SOD) activity, as well as increased proinflammatory molecules such as TNFα, IL1β, and IL6 [45]. All these changes may have negative effects on the brain-liver circuit responsible for the modulation of glucose metabolism by macronutrients. In this regard, these studies also found that a high-fat diet produced an increase in the content and the activity of adenosine monophosphate-dependent kinase (AMPK), which we had previously shown to attenuate the hypothalamic sensing and glucoregulatory action of leucine [22]. Recent studies in mice have suggested that insulin receptor (IR) signaling in Glut4 neurons was required to integrate hormonal and nutritional cues for the regulation of glucose metabolism [48]. Whether or not these Glut4 neurons play a role in the attenuation of nutrient sensing induced by HFD is currently unknown. Interestingly, a promising recent study in genetically modified mice showed that Gpr17 (a G protein-coupled receptor) deficiency in POMC neurons of the ARC, contributed to ameliorate metabolic alterations caused by long-term HFD feeding, at least in females [49]. Once again, it remains to be elucidated if the above mechanism would be helpful to reverse the attenuation of hypothalamic nutrient sensing caused by HFD feeding (Figure 1). In summary, further studies are required to better understand the mechanisms by which the consumption of high-fat diets interfere with hypothalamic regulation of liver glucose metabolism. Furthermore, it is conceivable that molecular interventions that curb the inflammatory processes in the hypothalamus may alleviate and contribute to restore the functionality of the nutrient sensing mechanisms controlling the circulating levels of glucose. This idea requires further experimental support. Interestingly, several natural products of vegetal origin hold promise in this potential therapeutic action. A compound that has received widespread attention is the polyphenol resveratrol, abundant in grapes and red wine [50,51]. In this regard, we and others have shown that direct administration of resveratrol in the hypothalamus stimulates hepatic glucose metabolism, resulting in a decrease in hepatic glucose production [52,53]. This effect is dependent on the activation of sirtuin 1 (Sirt-1), since the effect is abolished by siRNA mediated Sirt-1 attenuation [53]. Similar to resveratrol, other natural products of plant origin also possess activities that may be useful to ameliorate the inflammatory state of the hypothalamus.

## 6. Conclusions

Circulating macronutrients, such as amino acids, not only markedly influence insulin action, but they also serve as metabolic signals in the hypothalamus that contribute to the regulation of circulating glucose levels through changes in hepatic glucose metabolism. Signals that originate in CNS are relayed to the liver via the vagus nerve when the levels of macronutrients are high enough to activate a negative glucoregulatory mechanism in the MBH, causing an inhibition of glucose output by the liver. However, chronic consumption of high-fat and calorie-rich diets obliterates nutrient sensing, effectively conditioning a state of metabolic disability that makes the organism prone to developing hyperglycemia. In the long run, this acquired defect may contribute to the development of disease if measures are not taken to avoid further damage by these diets. Further studies are required to work out the detailed metabolic mechanisms leading to this condition.

## 7. Future Directions

Although we have learned a lot about the role of macronutrients in the central regulation of glucose metabolism and studies have shown that high-fat and calorie-rich diets attenuated the glucoregulatory action of circulating macronutrients, we still do not fully understand the mechanisms by which hypothalamic nutrient sensing is attenuated after chronic intake of HFD. Studies directed at identifying these mechanisms are key to developing strategies aimed at preventing and even overcoming this state of “metabolic disability”. In this regard, the recently postulated role of IR signaling on Glut4 expressing neurons in the hypothalamus [48], as mediators of the metabolic alterations brought about by HFD, deserves further study. Similarly, the observation that Gpr17 (a G protein-coupled receptor) deficiency in hypothalamic POMC neurons ameliorates the metabolic dysfunction caused by HFD is promising [49]. Studies aimed at modulating the activity of this receptor for restoring hypothalamic nutrient sensing should be performed. Lastly, studies that further explore the mechanistic details underlying the observed positive effects of leucine supplementation in overcoming the metabolic derangements of HFD consumption are also required. The studies just mentioned may have important implications for the treatment of metabolic diseases in humans.

## Data Availability

Not applicable.
